# Straw Retention with Reduced Fertilization Enhances Soil Properties, Crop Yields, and Emergy Sustainability of Wheat–Soybean Rotation

**DOI:** 10.3390/plants13131812

**Published:** 2024-07-01

**Authors:** Qi Yu, Xiaoying Jiao, Chenyu Wang, Yanbo Wang, Xiyang Xu, Zhenyuan Liu, Guangxin Ren, Yongzhong Feng

**Affiliations:** 1 College of Agronomy, Northwest A & F University, Xianyang 712100, China; 2 Shaanxi Engineering Research Center of Circular Agriculture, Xianyang 712100, China; 3 School of Science, Western Sydney University, Penrith, NSW 2751, Australia

**Keywords:** reduced fertilization, straw retention, soil quality, yield, emergy sustainability

## Abstract

Cereal + legume rotation is an integrated system that facilitates soil fertility and sustainable agricultural production. However, research on the management compatibility affecting soil physico-chemical properties yields overall agro-ecosystem sustainability, but profitability is lacking, especially under straw retention and potential reductions in fertilizer application. An 11-year field experiment investigated three treatments: no straw retention + traditional mineral fertilization (TNS), straw retention + traditional mineral fertilization (TS), and straw retention + reduced mineral fertilization (DS). Compared with TNS, TS significantly improved soil physico-chemical properties, including macro-aggregates (R > 0.25 mm), porosity, field water capacity (FWC), soil organic carbon (SOC) storage, total nitrogen storage, microbial biomass carbon (MBC), and microbial biomass nitrogen (MBN) by 17.3%, 3.2%, 13.0%, 5.5%, 3.2%, 15.5%, and 13.8%, respectively. TS also significantly increased total (wheat + soybean) yields (TYs), economic profits, and emergy sustainability index (ESI) by 15.8%, 25.0%, 3.7 times that of TNS, respectively. Surprisingly, compared with TS, DS further significantly improved R > 0.25 mm, porosity, FWC, SOC storage, MBC, MBN, TY, economic profits, and ESI by 11.4%, 1.5%, 6.1%, 3.0%, 10.6%, 7.2%, 5.7%, 11.1%, and 36.5%, respectively. Overall, retaining straw with reduced fertilization enhances soil properties, yields, and emergy sustainability in wheat–soybean rotation systems.

## 1. Introduction

The combined stress of soil degradation, need for food security, and global climate change have forced us to consider converting from conventional agriculture to sustainable agriculture [1,2]. Crop rotation can effectively enhance the utilization of agricultural resources (such as the land, water, and sunlight) in both temporal and spatial dimensions [3], which can also increase crop yields and ecological sustainability [4,5]. Therefore, it is widely recommended as an important measure for sustainable agricultural production [6,7] to achieve the United Nations Sustainable Development Goals (SDGs) [8]. Currently, the inclusion of legumes in cereal rotations not only improves soil quality by utilizing the ‘nutrition effect’ of leguminous crops [9], but also may reduce soil degradation caused by long-term use of double-gramineous rotation, mainly wheat (*Triticum aestivum* L.)–maize (*Zea mays* L.) rotation [10,11]. Moreover, cereal + legume rotation can also ensure food security because there is still a significant shortage of soybeans (*Glycine max* L.) in China’s food supply [12]. Therefore, exploring how to promote the sustainability of cereal + legume rotation through corresponding suitable production management practices is worth our consideration.

Crop straw and mineral fertilizer management practices are key factors affecting sustainable agricultural production [13]. Straw retention is an effective method of disposing large amounts of crop straw into the field compared with habitual removal or burning by farmers [14,15]. It can also be a substitute for mineral fertilizer and an economical way to trade off the excessive inputs of mineral fertilizer [16,17]. Studies related to the wheat and maize production system have demonstrated that straw retention can reduce soil bulk density (BD) and increase soil porosity and soil water content [18,19,20]. It can also result in the improvement of soil macro-aggregates (R > 0.25 mm) and mean weight diameter (MWD) [21,22,23]. Moreover, researchers have also found that straw retention can increase soil microbial populations in soil [24] and also increase the content of soil microbial biomass carbon (MBC), soil microbial biomass nitrogen (MBN), soil organic carbon (SOC), and soil total nitrogen (TN) [25,26,27,28,29]. Similarly, straw retention can increase grain yields, but reduced mineral fertilizer can also provide a sustainable yield and more economical profits [30,31]. Recent studies have also found straw retention in the cereal + legume rotation system is favorable for soybean crop growth (seed emergence, roots, and nodulations) and increases the leaf area index and soybean yield (by 4.4–68.3%) in northern India [32]. Also, straw retention could increase SOC (by 6.2%) at 0–20 cm depth in a 6-year maize–soybean rotation system in northeast China [33]. However, the differentiation in the regional climatic attributes (especially temperature and rainfall), local farming practices (such as crop and mineral fertilizer type), and the experimental duration (a short or a long term) are significant contributors in the agricultural production. However, under such circumstances, it is still unclear whether straw retention practices affect soil physical and chemical properties and how it can affect crop yields and economic benefits under long-term wheat–soybean rotation systems.

In addition, the agricultural ecosystem is a ‘nature, ecology, and economic’ complex system [34]. Therefore, we should not only focus on soil properties and crop yields, but also focus on the degree of dependence on the environment and the sustainability of the production system. The emergy evaluation method can analyze the structure, function, and quantify the value of the resource and environment of the agricultural ecosystem [35,36]. The emergy evaluation can strengthen people’s awareness towards sustainable and profitable agricultural management practices, which can also have a scientific significance in reasonably utilizing natural resources [37,38], and helps to formulate the national and regional agricultural policies under the umbrella of the UN SDGs [39]. Moreover, some important emergy evaluation indicators, such as the emergy self-sufficiency ratio (ESR), net emergy yield ratio (EYR), environmental loading ratio (ELR), and emergy sustainability index (ESI), can help to sustain/clear the agricultural production system. Moreover, it can also evaluate the degree of dependence on the environment, efficiency of resource utilization, production and economic pressure by agricultural activities, and overall sustainable development ability of the system. However, to date, there is little information on emergy evaluations under straw retention and reduced mineral fertilization management in a long-term wheat–soybean rotation system.

We hypothesized that straw retention with reduced fertilization could enhance soil properties, crop yields, economic profits, and overall sustainability of the wheat–soybean rotation system. Therefore, the specific objectives of this study were to evaluate the straw retention and reduced mineral fertilizer application effects on the following: (i) soil physical properties, which include R > 0.25 mm, MWD, soil porosity, BD, field water capacity (FWC), and saturated water capacity (SWC); (ii) soil chemical properties, which include C/N ratio, SOC storage, TN storage, and MBC and MBN content; and (iii) crop yields, economic profits, and emergy indicators (ESR, EYR, ELR, and ESI) in a long-term wheat–soybean rotation.

## 2. Results

### 2.1. Soil Physical Properties

**Figure 1:**
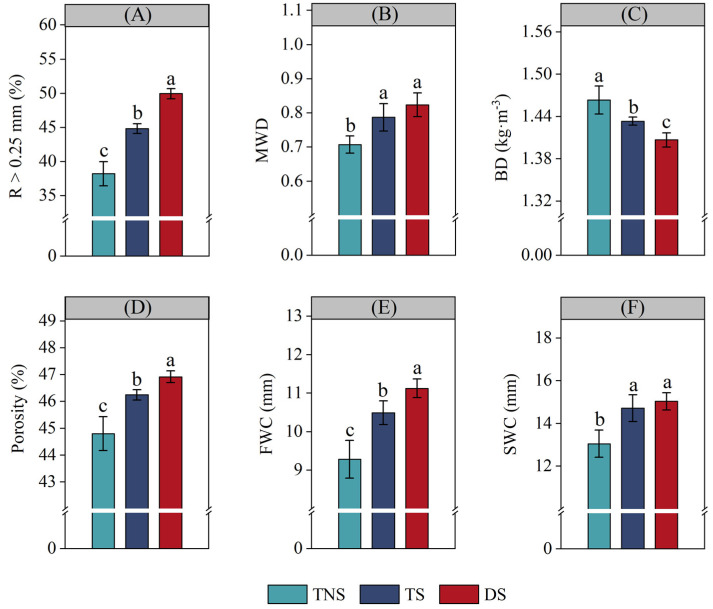
Soil physical properties of experimental treatments. TNS = no straw retention + traditional mineral fertilization; TS = straw retention + traditional mineral fertilization; DS = straw retention + reduced mineral fertilization, respectively. R > 0.25 mm = soil macro-aggregates; MWD = mean weight diameter of soil aggregate; BD = soil bulk density; FWC = field water capacity; SWC = saturated water capacity, respectively. The different lowercase letters indicate significant differences between treatments at the *p* < 0.05 level.

Compared with the TNS treatment, TS significantly increased R > 0.25 mm, MWD, porosity, FWC, and SWC by 17.3% (Figure 1A), 11.3% (Figure 1B), 3.2% (Figure 1D), 13.0% (Figure 1E), and 12.7% (Figure 1F), respectively, and significantly decreased BD by 2.1% (Figure 1C) (*p* < 0.05). Moreover, compared with TS, DS significantly increased R > 0.25 mm, porosity, and FWC by 11.4% (Figure 1A), 1.5% (Figure 1D), and 6.1% (Figure 1E), respectively, and significantly decreased BD by 1.9% (Figure 1C) (*p* < 0.05). Moreover, there were no differences in either MWD (Figure 1B) or SWC (Figure 1F) observed between the treatments of DS and TS.

### 2.2. Soil Chemical Properties

**Figure 2:**
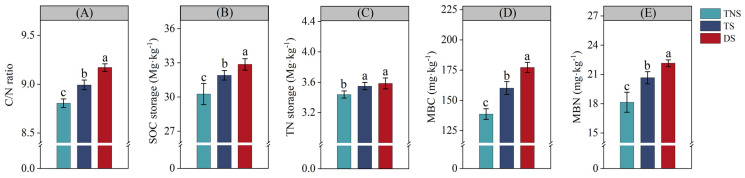
Soil chemical properties of experimental treatments. TNS = no straw retention + traditional mineral fertilization; TS = straw retention + traditional mineral fertilization; DS = straw retention + reduced mineral fertilization, respectively. C/N ratio = ratio of soil organic carbon/soil total nitrogen content; SOC = soil organic carbon; TN = soil total nitrogen; MBC = soil microbial biomass carbon; MBN = soil microbial biomass nitrogen, respectively. The different lowercase letters indicate significant differences between treatments at the *p* < 0.05 level.

Compared with the TNS treatment, TS significantly increased the soil C/N ratio, SOC storage, TN storage, MBC, and MBN by 2.1% (Figure 2A), 5.5% (Figure 2B), 3.2% (Figure 2C), 15.5% (Figure 2D), and 13.8% (Figure 2E) (*p* < 0.05), respectively. Moreover, compared with the TS treatment, DS significantly increased the soil C/N ratio, SOC storage, MBC, and MBN by 2.0% (Figure 2A), 3.0% (Figure 2B), 10.6% (Figure 2D), and 7.2% (Figure 2E) (*p* < 0.05), respectively. Moreover, there was no difference in TN storage (Figure 2C) observed between the treatments of DS and TS.

### 2.3. Yields and Economic Profits

For crop yields (Table 1), compared with the TNS treatment, TS significantly increased WY, SY, and TY by 11.9, 22.7, and 15.8% (*p* < 0.05), respectively. Moreover, compared with the TS treatment, DS significantly increased SY and TY by 11.2 and 5.7%, respectively (*p* < 0.05). No difference in WY was observed between DS and TS. In terms of economic profits (Table 1), compared with the TNS treatment, TS significantly increased WY, SY, and TY by 18.5, 29.3, and 25.0% (*p* < 0.05), respectively. In addition, compared with the TS treatment, DS significantly increased WY, SY, and TY by 6.0, 14.2, and 11.1% (*p* < 0.05), respectively.

### 2.4. Emergy Inputs, Outputs, and Emergy-Based Indicators

**Figure 3:**
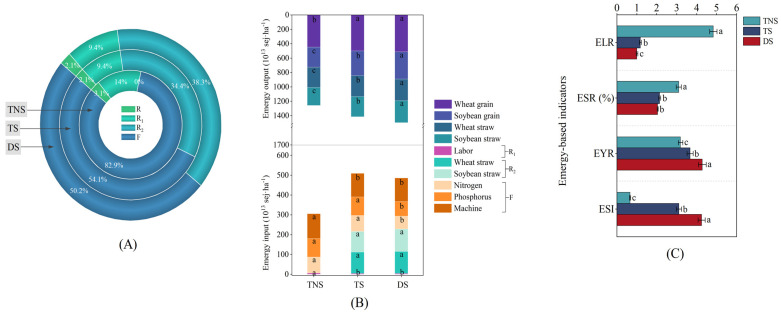
Distribution of emergy items (**A**); emergy inputs and outputs (**B**); and emergy-based indicators (**C**) of the experimental treatments. TNS = no straw retention + traditional mineral fertilization; TS = straw retention + traditional mineral fertilization; DS = straw retention + reduced mineral fertilization, respectively; R = renewable natural resources; R_1_ = renewable organic energy; R_2_ = system feedback energy; F = non-renewable industrial auxiliary energy; ELR = environmental loading ratio; ESR = emergy self-sufficiency ratio; EYR = net emergy yield ratio; ESI = emergy sustainability index, respectively. The different lowercase letters indicate significant differences between treatments at the *p* < 0.05 level.

The emergy inputs included R, R_1_, R_2_, and F (Figure 3A), and the three treatments (TNS, TS, and DS) had the same percentage of R. Compared with the TNS treatment, TS decreased the percentage of R_1_ and F and increased the percentage of R_2_. Moreover, compared with the TS treatment, DS increased the percentage of R_2_ and decreased the percentage of F. In detail, regarding the changes in emergy inputs (Figure 3B, the lower part), compared with the TNS treatment, TS significantly decreased labor by 43.5% and machine operation by 5.7% and significantly increased wheat straw by 1.1 times and soybean straw by 1.0 times (*p* < 0.05). Moreover, compared with the TS treatment, DS significantly decreased the emergy inputs of nitrogen by 25.0% and phosphorus by 26.3% (*p* < 0.05). There was no difference in the emergy inputs among labor, wheat straw, soybean straw, and machine operation between the treatments of DS and TS. In addition, emergy outputs (Figure 3B, upper part) comprised wheat grain, soybean grain, wheat straw, and soybean straw. Compared with the TNS treatment, TS significantly increased wheat grain, soybean grain, wheat straw, and soybean straw by 11.9, 22.7, 6.4, and 12.6% (*p* < 0.05), respectively. Moreover, compared with the TS treatment, DS significantly increased the emergy outputs of soybean grain, wheat straw, and soybean straw by 11.2, 3.0, and 8.8% (*p* < 0.05), respectively. There was no difference in the emergy outputs of wheat grain between the treatments of DS and TS.

For emergy-based indicators (Figure 3C), compared with the TNS treatment, TS significantly decreased the environmental loading ratio (ELR) by 3.1 times and emergy self-sufficiency ratio (ESR) by 49.9% and significantly increased the net emergy yield ratio (EYR) by 15.1% and emergy sustainability index (ESI) by 3.7 times (*p* < 0.05). Moreover, compared with the TS, DS significantly decreased the ELR by 17.0% and significantly increased the EYR by 16.7% and the ESI by 36.5% (*p* < 0.05).

### 2.5. Relationships between Yields, Soil Physical, and Chemical Properties

**Figure 4:**
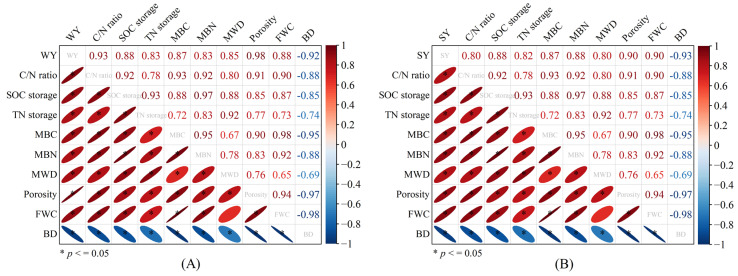
Correlationships between soil physical, chemical properties, and yields (wheat, **A**; Soybean, **B**). WY = wheat grain yield; C/N ratio = ratio of soil organic carbon/soil total nitrogen; SOC = soil organic carbon; TN = soil total nitrogen; MBC = soil microbial biomass carbon; MBN = soil microbial biomass nitrogen; MWD = mean weight diameter of soil aggregate; FWC = field water capacity; BD = soil bulk density, respectively. The symbol ‘*’ denotes significant differences between treatments at the *p* < 0.05 level.

A correlation analysis showed that WY (Figure 4A) and SY (Figure 4B) were both significantly correlated with soil physical (MWD, porosity, FWC, and BD) and chemical properties (C/N ratio, SOC storage, TN storage, MBC, and MBN content) (*p* < 0.05). Moreover, the correlation coefficients between WY and soil physical and chemical properties were ranked as follows (Figure 4A): porosity (0.98) > C/N ratio (0.93) > FWC (0.88) > SOC storage (0.88) > MBC (0.87) > MWD (0.85) > MBN (0.83) > TN storage (0.83) > BD (−0.92). The correlation coefficients between SY and soil physical and chemical properties were ranked as follows (Figure 4B): FWC (0.90) > porosity (0.90) > MBN (0.88) > SOC storage (0.88) > MBC (0.87) > TN storage (0.82) > C/N ratio (0.80) > BD (−0.93). In addition, all indices had a significant positive relationship with each other, except for the relationship between FWC and MWD, and there was a significant negative relationship between BD and other indices.

## 3. Discussion

### 3.1. Effects of Treatments on Soil Physical Properties

In an 11-year field experiment, our study found that TS significantly increased the number of soil macro-aggregates (R > 0.25 mm) and mean weight diameter (MWD) compared with TNS (Figure 1A,B). This may be attributed to crop straw retention practice, because straw contains abundant organic and a few inorganic elements [13], benefits the increase in soil organic matter content [40], and further plays a ‘glue’ role in soil macro-aggregate formation [41] and aggregate stability [42]. And this could be confirmed in our study, since TS significantly improved both MBC content and SOC storage when compared with TNS (Figure 2B,D), which benefited the enhancement of macro-aggregates. Moreover, there exists a significant correlation between MBC, SOC storage, and MWD (Figure 4).

In addition, we found that TS significantly reduced BD and significantly increased soil porosity, FWC, and SWC compared with TNS (Figure 1C–F). This may be because straw retention forms a barrier of ‘soil–straw–atmosphere’ that may prevent direct wind and rainfall erosion [43], which helps to reduce soil structural compaction [44,45]. At the same time, this barrier can also slow down the soil evaporation rate [46,47], which increases the soil’s water-holding capacity [48,49]. Furthermore, DS significantly decreased BD and significantly increased R > 0.25 mm, porosity, and FWC compared with TS (Figure 1A,C–E). This may be attributed to reduced mineral fertilization under straw retention practices, which alleviates secondary soil salination and has a negative impact on soil bulk density, porosity, and soil microorganisms’ reproduction [50], benefitting the maintenance of soil structure and soil water capacity.

These results agree with our hypothesis that the retention of straw in the field and the potential reduction in mineral fertilizer application following straw retention may improve soil physical properties, such as soil porosity, BD, R > 0.25 mm, MWD, FWC, and SWC in wheat–soybean rotation systems, especially DS treatment. Moreover, in our future studies, we will also focus on the aspects of soil temperature, pH, and greenhouse gas (CO_2_, CH_4_, and N_2_O) emissions, and look to clarify how straw retention and mineral fertilization with straw retention management impact the carbon footprint and the key impact factors, under a wheat–soybean rotation production system.

### 3.2. Effects of Treatments on Soil Chemical Properties

In an 11-year experiment, we found that TS significantly increased the soil C/N ratio, SOC storage, TN storage, MBC content, and MBN content compared with TNS (Figure 2). There are two mechanisms involved: the first is that crop straw retention can improve soil physical properties, such as R > 0.25 mm, MWD, soil porosity, FWC, and SWC (Figure 1). These soil physical properties provide a favorable condition for soil organic carbon and nitrogen storage, which was also confirmed by the previous studies [51,52]. And secondly, wheat straw retention increased the soil C/N ratio due to its high carbon and low nitrogen content [53], which promoted available nitrogen to be assimilated into the soil nitrogen pool [54,55]. Similarly, we also observed an increase in the MBN and MBC content, and a higher content of MBN and MBC with more efficient resource utilization [56] and a higher nutrient supply for maintaining SOC and TN storage [57]. Moreover, soybean straw has a high nitrogen content and low C/N ratio [58] and is easily decomposed by microorganisms [28,59], and returning soybean straw into soil may improve SOC and TN storage [45].

In addition, we also found that DS significantly increased the soil C/N ratio, SOC storage, and MBC and MBN content compared with the TS treatment (Figure 2A,B,D,E). This may be attributed to improved soil physical properties (Figure 1), and it further provided relatively enough soil aeration and water conditions for soil microorganisms’ reproduction, thereby helping to increase the soil C/N ratio, SOC storage, and MBC and MBN content [60]. Moreover, as an economical green manure crop, soybean can increase the soil nitrogen content due to its biological nitrogen fixation capacity [10], and during their growth process, soybean crops form a large amount of dead root nodules and sediment, which can be an important nitrogen source for subsequent crop utilization and help to save mineral fertilizers [49,61]. As the study results showed, under the condition of no nitrogen fertilizer application, the biological nitrogen fixation efficiency of soybeans under normal rainfall levels was 47–70% in China [62], which could benefit to reduce mineral fertilizations, especially saving nitrogen fertilizers. But we need focus on the biological nitrogen fixation efficiency of soybeans in our study region in the future, because there are still significant differences in different production areas.

These results agree with our hypothesis that retention of straw in the field and the potential reduction in mineral fertilizer application following straw retention may ameliorate soil chemical properties, such as the soil C/N ratio, SOC storage, TN storage, and MBC and MBN content, which is consistent with the results of previous studies [63,64]. Moreover, we also provide evidence that straw retention practices can moderately reduce mineral fertilizer inputs and improve soil chemical properties in a wheat–soybean rotation production system, but this should be further confirmed under different conditions, such as initial soil physiochemical properties, climatic conditions, fertilization type and intensity, and study regions [65]. The continuous exploration of the differences in straw retention with mineral fertilizer management and the effects thereof on soil chemical properties in different soil layers requires focus in future research. Furthermore, enzyme activity indicators related to carbon and nitrogen cycling should be analyzed, and microbial functional genes should be studied to explain the improvements in soil chemical properties from a mechanistic level to enhance the sustainability of wheat–soybean rotation.

### 3.3. Effects of Treatments on Crop Yields, Economic Profits, and Emergy Evaluation

Crop yields are a comprehensive reflection of soil productivity [66]. In an 11-year wheat–soybean rotation field experiment, we found that TS and DS significantly increased the wheat, soybean, and total (wheat + soybean) yield compared with TNS (Table 1). This may be attributed to both the soil physical properties (including R > 0.25 mm, MWD, soil porosity, FWC, and SWC) (Figure 1) and soil chemical properties (including soil C/N ratio, SOC storage, TN storage, and MBC and MBN content) (Figure 2), which were improved under the TS and DS treatments. These properties may create a relatively suitable environment for soil moisture, ventilation, and nutrients [67,68] that are good for crop growth and root development [69], as well as provide adequate and effective soil nutrients that increase yield [9,70]. These relationships between crop yields and soil physical and chemical properties [71] were confirmed by the correlation analysis in our study (Figure 4). Moreover, a long-term field experiment may also play an important role in improving these soil physical and chemical properties, further maintaining crop yields [72,73]. At the same time, obtaining as much economic profit as possible is an important prerequisite for the profitability of agricultural producers and the sustainable development of crop production systems [74]. The results for crop yields and the explanations for the yield increase are well explained: TS significantly increased the economic profits of wheat, soybean, and their combination (wheat + soybean) compared with TNS, and DS significantly increased the economic profits of wheat, soybean, and their combination (wheat + soybean) compared with TS (Table 1).

Except for focusing on the crop yields and economic profits in the wheat–soybean rotation system, the boundary issues of the agricultural ‘production, ecology, and economy’ composite system can be comprehensively considered through emergy evaluation [75,76]. We found that TS significantly decreased the ELR and ESR and significantly increased the EYR and ESI compared with TNS (Figure 3C). This may be attributed to straw retention practices, which can decrease the renewable organic energy (R_1_) by significantly decreasing the emergy input of the labor and increasing the non-renewable industrial auxiliary energy (F) by significantly decreasing the emergy input of agricultural machinery, increasing the system feedback energy (R_2_), significantly increasing the emergy input of wheat and soybean straw, and increasing emergy outputs compared with straw removal treatment (Figure 3B). Moreover, we found that DS significantly decreased the ELR and ESR and increased the EYR and ESI compared with TS (Figure 3C), which may be attributed to reduced mineral fertilizer application under straw retention practices. This reduced F by significantly decreasing the emergy input of nitrogen and phosphorus and significantly increasing the emergy output (soybean grain, wheat straw, and soybean straw) (Figure 3B).

These results agree with our hypothesis that retention of straw in the field and the potential reduction in mineral fertilizer application following straw retention may enhance crop yields, economic profits, and emergy sustainability. The results show the effect of straw retention and mineral fertilizer management on crop yields and economic profits in a wheat–soybean rotation system. They also provide decision support based on emergy evaluations, which helps to increase the efficiency of resource utilization, reducing the pressure and dependence of agricultural food and economic production activities on the natural environment and promoting sustainable development in a wheat–soybean rotation system.

## 4. Materials and Methods

### 4.1. Field Experiment Description

The field experiment was initiated in September 2010. The experiment was located at the Northwest A&F University Field Experimental Station (34°12′ N, 108°07′ E), Shaanxi Province, China. The site has an average annual temperature of 12.9 °C, precipitation of 660.3 mm, and solar radiation of 2163.4 h (it belongs to the continental warm, temperate, and monsoonal climate). Study plots were flat with Lou soil, and the texture was silt clay loam [77]. The basic soil nutrient content at 0–40 cm depth was as follows: 7.0 g·kg^−1^ SOC, 0.7 g·kg^−1^ TN, 0.5 g·kg^−1^ soil total phosphorus, and 136.3 mg·kg^−1^ available potassium.

### 4.2. Experimental Design

The field experiment was set up in a split plot design with three replicates, comprised of nine plots (8.6 × 8 m) in total. The treatments were as follows: no straw retention + traditional mineral fertilization (TNS, as a control), straw retention + traditional mineral fertilization (TS), and straw retention + reduced mineral fertilization (DS). The treatment of traditional mineral fertilizer means the number and type of mineral fertilizers by local farmers commonly used; the treatment of reduced mineral fertilizer means a 20% reduction in mineral fertilization on the basis of the traditional mineral fertilization treatment.

The major local varieties of wheat (xinong889) and soybean (dongdou339) seeds were the same across the three treatments. The national approval number was 2005001 and the breeding unit for the variety was the experimental farm of Northwest A&F University for wheat (xinong889). Moreover, the national approval number was 2008019 and the breeding unit for the variety was Liaoning Dongya Seed Industry Co., Ltd. (Shenyang, China). for soybeans (dongdou339). The wheat was sown and harvested in mid-October and early June annually, and the soybean was sown after the wheat was harvested and was harvested in late September. The time interval between the wheat harvest and soybean sowing was usually within ten days. Details of the annual mineral fertilizer application amount (before sowing) and straw management (after harvesting) are shown in Table 2. During the field experiment, conservation tillage and no irrigation were adopted. Other agricultural practices, such as weed and pest control after harvest, complied with local farming practices.

### 4.3. Soil Sampling and Analysis

Bulk soil samples were sampled randomly at five points using a hand auger from the topsoil (0–20 cm) layer after the soybean was harvested in late September 2021. BD was measured from an undisturbed soil sample plot (60 cm depth × 60 cm length × 40 cm width) using the metal ring (100 cm^3^) method [78]. Once collected, samples were thoroughly mixed and all visible stubble, stones, and roots were removed manually. Samples were then sieved (2 mm) and split into two parts: one sub-sample was stored at −20 °C, and the other sub-sample was air-dried at room temperature for subsequent laboratory analysis. Subsamples for soil aggregates were collected after wheat harvesting, and soil aggregate size-class proportion analysis adopted the wet-sieving method [79]. The sizes of macro-aggregates (R > 0.25 mm) and micro-aggregates (R < 0.25 mm) were obtained. The MWD was calculated following Fungo’s (2017) description [80]. FWC, SWC, and soil porosity were calculated following Long’s (2023) description [81]. Moreover, after the soil samples were air-dried and finely ground through a 0.25 mm sieve, SOC and TN content were determined by adopting the K_2_Cr_2_O_7_–H_2_SO_4_ digestion and Kjeldahl methods, respectively [26]. SOC and TN storage were calculated based on the equivalent soil mass method [82]. MBC and MBN were measured and calculated according to Bao’s (2000) description [83].

Wheat grain yield (WY) and soybean grain yield (SY) were determined by hand harvesting, threshing, and air-drying (a water content of 13%) from the 1 m^2^ sampling quadrat, with three replicates. The total grain yield (TY) was the sum of the wheat and soybean grain yields annually. Economic profits were calculated by multiplying the WY and SY by the average market price (from the years 2019 to 2023) of both the wheat (2.4 CNY/kg) and soybean (4.9 CNY/kg), respectively, over 5 years [84]. The total economic profits were the sum of the economic profits of wheat and soybean annually.

### 4.4. Emergy Evaluation Method

The inputs and outputs of the wheat–soybean rotation system and the local environmental resources (including solar radiation, wind, and rainfall) were recorded within the experimental years and considered in the emergy evaluation analysis. By applying H. T. Odum’s ‘Energy System Language’ Legend, an energy diagram (the functional unit was per hectare) was drawn [85], and the main characteristics of both the straw retention (Figure S1A) and no straw retention (Figure S1B) experimental systems were identified.

Moreover, the emergy driving the experimental system was divided into four types: renewable natural resources (R), renewable organic energy (R_1_), system feedback energy (R_2_), and non-renewable industrial auxiliary energy (F). The energy value of each type was calculated by multiplying the local resource input amount and the energy coefficients [86]. Then, the energy was converted into emergy values (sej/ha) using multiple transformity factors (UEV), which were obtained from published studies, as shown in Table S1.

The global emergy baseline was set as 15.83 × 10^24^ sej/year in the study according to Odum’s description [87]. Moreover, in accordance with emergy algebra rules, avoiding double accounting, only the maximum rainwater chemical energy in R was considered [85]. Only 37% of the emergy input required for the recycling of wheat and soybean straw was assumed to be renewable in R_2_ [75]. To evaluate the sustainable development performance of the three treatments, the emergy-based indicators were calculated (including emergy self-sufficiency ratio, ESR; environmental loading ratio, ELR; net emergy yield ratio, EYR; and emergy sustainability index, ESI), and the items, expressions, and meanings are shown in Table S2.

### 4.5. Statistical Analyses

Excel 2016 and the SPSS statistical package v.19.0 (SAS Inst., located in Cary, NC, USA) were utilized for data analysis and statistical tests following an 11-year experiment with three treatments. Figures were created using Origin 2023. Significance was determined through an analysis of variance (ANOVA), and the least significant difference (LSD) method was employed to compare the differences among the treatment means (significance denoted by ‘*’ at *p* < 0.05, and ‘**’ at *p* < 0.01).

## 5. Conclusions

In our 11-year field experiment, the retention of crop straw in the field (TS treatment) significantly improved soil physical properties (including R > 0.25 mm, MWD, BD, porosity, FWC, and SWC) and soil chemical properties (including C/N ratio, SOC storage, TN storage, and MBC and MBN content) and significantly increased yields (wheat and soybean) and total economic profits compared with the no straw retention treatment in a wheat–soybean rotation. Moreover, these soil properties, yields, and economic profits were significantly enhanced under the straw retention practice with reduced mineral fertilizer management (DS treatment). Moreover, the DS treatment also increased the sustainability of the wheat–soybean rotation system by significantly decreasing the ELR and ESR and increasing the EYR and ESI when compared with the TS treatment. Therefore, retaining crop straw with reduced mineral fertilization management (DS) could represent a promising method for improving soil quality and increasing crop yields, economic profits, and sustainability in wheat–soybean rotation systems. This study has practical significance in the management of straw retention with mineral fertilization to achieve the economical profitability and sustainable production of wheat–soybean rotations in China.

## Figures and Tables

**Table 1:** Grain yields and economic profits of experimental treatment.

Treatment	Grain Yields (kg·ha^−1^)			Economic Profits (CNY·ha^−1^)		
	Wheat (WY)	Soybean (SY)	Wheat + Soybean (TY)	Wheat (WEP)	Soybean (SEP)	Wheat + Soybean (TEP)
TNS	4183.7 b	2409.1 c	6592.8 c	6190.5 c	9330.9 c	15,521.4 a
TS	4680.5 a	2956.4 b	7636.9 b	7333.6 b	12,067.2 b	19,400.8 b
DS	4788.9 a	3286.9 a	8075.8 a	7771.5 a	13,783.7 a	21,555.2 a

Note: TNS = no straw retention + traditional mineral fertilization; TS = straw retention + traditional mineral fertilization; DS = straw retention + reduced mineral fertilization, respectively. WY = wheat grain yield; SY = soybean grain yield; TY = total (soybean + wheat) grain yield; WEP = wheat economical profits; SEP = soybean economical profits; TEP = total (soybean + wheat) economical profits, respectively. The different lowercase letters indicate significant differences between treatments at the *p* < 0.05 level.

**Table 2:** Mineral fertilizer application amount and straw management.

Treatment	Mineral Fertilizer Application Amount (kg·ha^−1^)	Crop Straw Management (Both Wheat and Soybean)
TNS	Wheat: N = 111.1; P_2_O_5_ = 78.2 Soybean: N = 0; P_2_O_5_ = 32.2	All above ground straw was removed
TS	Wheat: N = 111.1; P_2_O_5_ = 78.2 Soybean: N = 0; P_2_O_5_ = 32.2	Above ground straw was crushed into 3–5 cm fragments and returned to field
DS	Wheat: N = 88.9; P_2_O_5_ = 62.6 Soybean: N = 0; P_2_O_5_ = 25.8	Same as the TS treatment above

Note: TNS = no straw retention + traditional mineral fertilization; TS = straw retention + traditional mineral fertilization; DS = straw retention + reduced (80% traditional) mineral fertilization, respectively.

## Data Availability

Data is contained within the article and Supplementary Materials.

## Supplementary Materials

The following supporting information can be downloaded at: https://www.mdpi.com/article/10.3390/plants13131812/s1, Figure S1: Emergy analysis diagram of wheat–soybean rotation system of straw and no straw with mineral fertilization; Table S1: The transformity factors of emergy analysis; Table S2: Emergy-based indicators calculation.
