# Supplementary material for: Straw Retention with Reduced Fertilization Enhances Soil Properties, Crop Yields, and Emergy Sustainability of Wheat–Soybean Rotation
Source: Plants (Basel). 2024 Jul 1;13(13):1812. doi: 10.3390/plants13131812 (PMC11244234; doi:10.3390/plants13131812)
Supplement: Supplementary file 1 [file plants-13-01812-s001.zip › plants-3053365-supplementary.pdf]

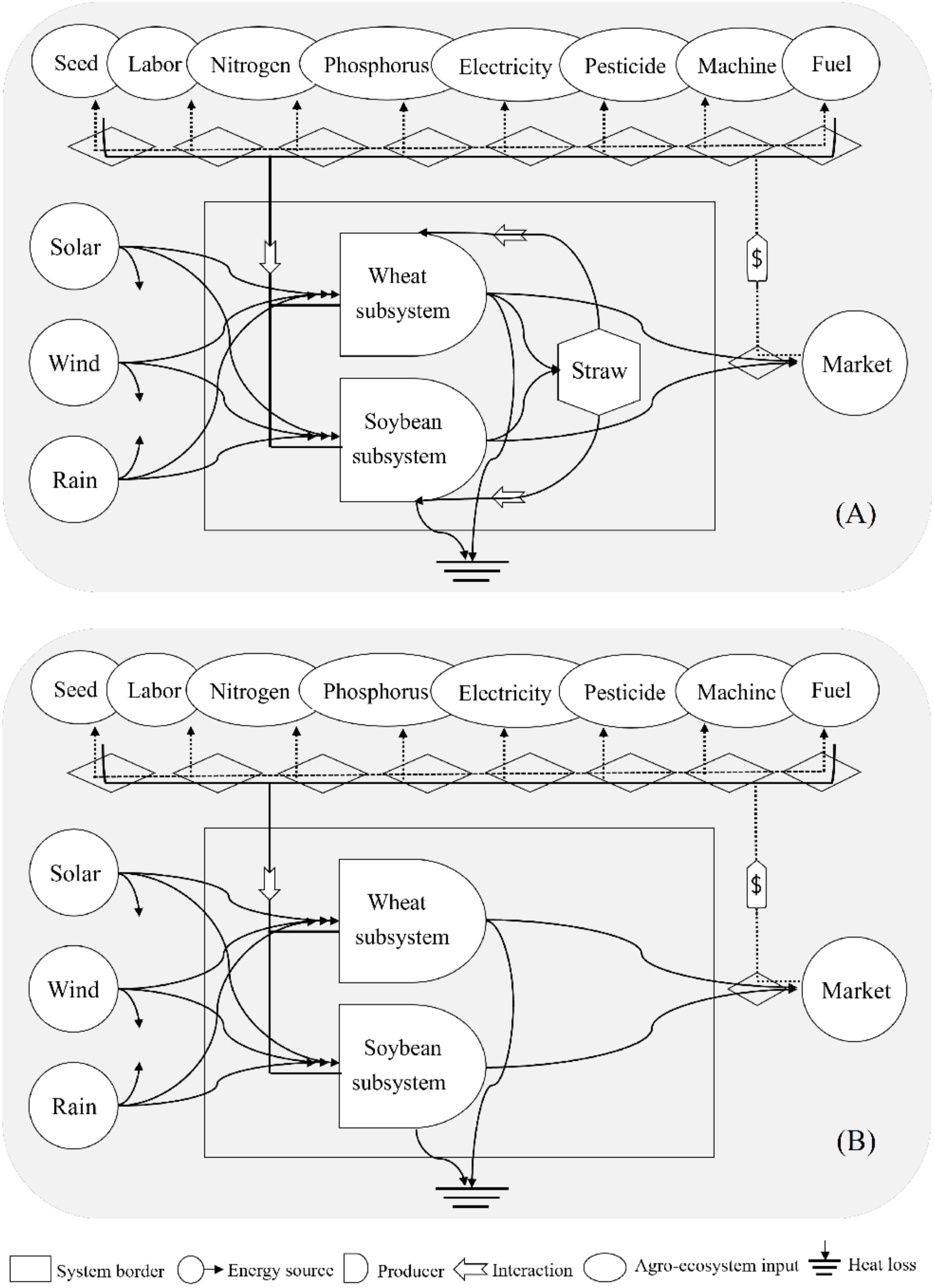

Figure S1. Emergy analysis diagram of wheat–soybean rotation system of straw and no straw with mineral fertilization.

Table S1. The transformity factors of emergy analysis.

Items	UEV (sej·J^{-1})	Reference
**Renewable natural resources (R)**		
Solar energy	1.00E+00	Odum et al. (1996)
Wind energy	6.63E+02	Lan et al. (2002)
Rainwater chemical energy	1.54E+04	Odum et al. (1996)
Rainwater potential energy	8.89E+03	Lu et (2005)
**Renewable organic energy (R_1)**		
Wheat seed	6.80E+04	Odum et al. (1996)
Soybean seed	7.65E+04	Ulgiati et al. (1993)
Labor	3.80E+05	Odum et al. (1996)
**System feedback energy (R_2)**		
Wheat straw	3.90E+04	Lan et al. (2002)
Soybean straw	3.90E+04	Lan et al. (2002)
**Non-renewable industrial auxiliary energy (F)**		
Nitrogen	4.62E+09	Lan et al. (2002)
Phosphorus	3.90E+09	Lan et al. (2002)
Pesticide	1.62E+09	Odum et al. (1996)
Machine	7.50E+07	Lan et al. (2002)
Fuel	6.60E+04	Odum et al. (1996)
Electricity	1.59E+05	Odum et al. (1996)

Table S2. Emergy-based indicators calculation.

Items	Expression	Meanings
Wheat grain yield (WY), kg/ha	WY	Wheat grain yield per kilogram per hectare
Soybean grain yield (SY), kg/ha	SY	Soybean grain yield per kilogram per hectare
Wheat straw yield (WSY), kg/ha	WSY	Wheat straw yield per kilogram per hectare
Soybean straw yield (SSY), kg/ha	SSY	Soybean straw yield per kilogram per hectare
Emergy inputs (T_1), sej/ha	$T_1 = R + R_1 + R_2 + F$	Total agricultural production input
Emergy outputs (T_2), sej/ha	$T_2 = WY + SY + WSY + SSY$	Total agricultural production output
Emergy self-sufficiency ratio (ESR), %	$ESR = T_1 / R$	The degree of dependence on the environment
Net-emergy yield ratio (EYR)	$EYR = T_2 / (R_1+F)$	The efficiency of resource utilization or market competitiveness
Environmental loading ratio (ELR)	$ELR = F / (R + R_1 + R_2)$	The pressure of economic production activities on the natural environment
Emergy sustainability index (ESI)	$ESI = EYR / ELR$	The sustainable development ability of experimental system

Supplementary materials

Title: Straw Retention with Reduced Fertilization Enhances Soil Properties, Crop Yields, and Emergy Sustainability of Wheat–Soybean Rotation

Author names: Qi Yu [1,2], Xiaoying Jiao [1,2], Chenyu Wang [1,2], Yanbo Wang [3], Xiyang Xu [1,2], Zhenyuan Liu [1,2], Guangxin Ren [1,2] and Yongzhong Feng [1,*]

Affiliation:

1. College of Agronomy, Northwest A & F University. Yangling 712100, Shaanxi, China

2. Shaanxi Engineering Research Center of Circular Agriculture, Yangling, 712100, Shaanxi, China

3. School of Science, Western Sydney University, Penrith 2751, NSW, Australia

*** Corresponding author:** Professor Yongzhong Feng

*** Corresponding author address:** College of Agronomy, Northwest A & F University. Yangling 712100, Shaanxi, China

Tel: +86-029-87080736; **E-mail address:** fengyz@nwsuaf.edu.cn

Supplementary text

Text 1. Emergy analysis diagram of wheat–soybean rotation system of straw and no straw with mineral fertilization.

By applying H. T. Odum’s Energy System Language Legend, an emergy diagram (with a functional unit per hectare) of the wheat–soybean rotation system was shown in Fig. S1, including straw retention (A) and no straw retention (B) with mineral fertilization. The wheat–soybean rotation system of straw retention, which border contains wheat subsystem, soybean system, and straw (Fig. S1A), and the wheat–soybean rotation system of no straw retention contains straw retention, which border contains wheat subsystem (Fig. S1B), respectively. Moreover, the energy sources (solar, wind, and rain), producers (comprising wheat and soybean

subsystems), and inputs to the agro-ecosystem (such as seeds, labor, nitrogen, phosphorus, electricity, pesticides, machinery, and fuel) were shown in Fig. S1.

Text 2. The transformity factors of emergy analysis.

The energy was converted into emergy values using multiple transformity factors (UEV). The four types of the emergy driving of the experimental system was contains renewable natural resources (R), renewable organic energy (R_1), system feedback energy (R_2), and non-renewable industrial auxiliary energy (F), and each type of the UEV of different items and reference were shown in Table S1, respectively.

Text 3. Emergy-based indicators calculation.

Emergy evaluation analysis indicators of emergy self-sufficiency ratio (ESR), net-emergy yield ratio (EYR), environmental loading ratio (ELR), and emergy sustainability index (ESI) were calculated, and the related items, expression, and meanings of them were shown in Table S2, respectively.
